# COMP report: CPQR technical quality control guidelines for CT simulators

**DOI:** 10.1002/acm2.12213

**Published:** 2017-11-16

**Authors:** Philippe Després, Stewart Gaede

**Affiliations:** ^1^ Département de radio‐oncologie CHU de Québec – Université Laval Québec QC Canada; ^2^ Département de physique, de génie physique et d'optique Université Laval Québec QC Canada; ^3^ Department of Oncology and Medical Biophysics Western University London ON Canada; ^4^ Department of Physics and Engineering London Regional Cancer Program London ON Canada

**Keywords:** computed tomography, quality assurance in radiotherapy, simulation

## Abstract

The Canadian Organization of Medical Physicists (COMP), in close partnership with the Canadian Partnership for Quality Radiotherapy (CPQR) has developed a series of Technical Quality Control (TQC) guidelines for radiation treatment equipment. These guidelines outline the performance objectives that equipment should meet in order to ensure an acceptable level of radiation treatment quality. The TQC guidelines have been rigorously reviewed and field tested in a variety of Canadian radiation treatment facilities. The development process enables rapid review and update to keep the guidelines current with changes in technology (the most updated version of this guideline can be found on the CPQR website). This particular TQC details recommended quality control testing of CT simulators.

## 1 INTRODUCTION

The Canadian Partnership for Quality Radiotherapy (CPQR) is an alliance among the three key national professional organizations involved in the delivery of radiation treatment in Canada: the Canadian Association of Radiation Oncology (CARO), the Canadian Organization of Medical Physicists (COMP), and the Canadian Association of Medical Radiation Technologists (CAMRT). Financial and strategic backing is provided by the federal government through the Canadian Partnership Against Cancer (CPAC), a national resource for advancing cancer prevention and treatment. The mandate of the CPQR is to support the universal availability of high quality and safe radiotherapy for all Canadians through system performance improvement and the development of consensus‐based guidelines and indicators to aid in radiation treatment program development and evaluation.

This document contains detailed performance objectives and safety criteria for *Computed Tomography simulators*. The development of the individual TQC guidelines is spearheaded by expert reviewers and involves broad stakeholder input from the medical physics and radiation oncology community.^1^

All information contained in this document is intended to be used at the discretion of each individual center to help guide quality and safety program improvement. There are no legal standards supporting this document; specific federal or provincial regulations and license conditions take precedence over the content of this document.

## 2 SYSTEM DESCRIPTION

The purpose of radiation planning simulation is to simulate as accurately as possible the patient's position, shape, and anatomy relative to the radiation therapy machine and isocenter.^2^, ^3^, ^4^ Modern treatment machines are able to achieve mechanical accuracies in the range of ±1 mm and ±1° and, so too, shall the simulators used to plan these radiation treatments. The process of radiation treatment planning frequently involves the following steps:

1. Acquisition of a volumetric computed tomography (CT) dataset;
2. Transfer of the CT dataset to a radiation treatment planning workstation;
3. Marking of patient‐based reference points before or after virtual beam planning;
4. Localization of targets and critical structures;
5. Virtual beam planning; and
6. Dose calculations.

For the purpose of this document, steps 1, 2, and 3 define the process of CT simulation. Steps 1, 2, 3, and sometimes 4, occur with the patient present in the CT scanner room.

CT simulators consist of a state‐of‐the‐art spiral (or helical) CT scanner,^5^, ^6^ the associated acquisition/processing computer system, a patient laser marking system, and radiation treatment accessories. CT images provide the anatomical, geometrical, and relative electron density information necessary for the precision radiation planning. The CT computer is networked to a 3D virtual simulation workstation or full radiation treatment planning (RTP) system. These workstations provide software tools for the localization of the targets, co‐registration of the CT images with other imaging modalities, graphical planning of the radiation beams, and the production of digitally reconstructed radiographs (DRRs) in a beam's eye view (BEV). The difference between 3D virtual simulation workstations and full RTP systems is the dose calculation and dose evaluation capabilities that are integral with the latter. The process of CT simulation has been described in detail by various authors.^2^, ^3^, ^4^

For CT simulators, tests are required for optical, mechanical, radiographic, and safety systems. The standards for CT simulator quality control are listed in the tables below. These standards consist of tests to be performed, along with their minimum frequency. The tests are derived from the published literature and, in particular, the standards laid out in the American Association of Physicists in Medicine (AAPM) TG‐40 document,^7^ the AAPM TG‐66 document,^8^ the Institute of Physics and Engineering in Medicine (IPEM) document, Report 81,^9^ the Health Canada Safety Code 35,^10^ and other resources providing further information on tests and CT characteristics.^3^, ^11^, ^12^

Included in the scope of this document is four‐dimensional computed tomography (4D‐CT), which has been developed to characterize 3D volumes of a patient's thorax and/or abdomen during respiration with reduced artifacts. This requires the acquisition of multiple projections of the same volume during free breathing and sorting either the projection data (sinogram space) or reconstructed axial slices (image space) according to the respiratory phase monitored simultaneously during the CT scan. CT acquisition can be acquired in cine mode, where the couch is fixed during scanning, or in low‐pitch helical mode. With the implementation of multislice CT scanners, the pitch can be low enough to allow for sampling the anatomy over a respiratory cycle. The sorting of the CT data is guided by a respiratory trace. The most common approaches to reconstruct 4D‐CT datasets involve the use of chest/abdominal marker displacement, strain gauge, or spirometry. Despite the variety of 4D‐CT reconstruction and re‐sorting algorithms, the resulting CT dataset is typically composed of 8 to 10 3D‐CT datasets corresponding to different phases of the respiratory cycle. The encompassing volume of a target can then be produced from a 4D‐CT dataset providing an accurate representation of the tumor volume due to respiratory motion during radiation delivery. A subset of the 4D‐CT dataset can also be used for respiratory‐gated radiotherapy where the radiation beam is triggered only during a preselected portion of the respiratory cycle.

Routine quality assurance involves the use of programmable respiratory motion phantom(s). As 4D‐CT reconstruction strategies vary from vendor to vendor, the ability to routinely reconstruct the 3D images of a known object of known geometry, electron density, amplitude, and period into the desired number of respiratory phases, form the basis of routine quality assurance of 4D‐CT imaging. Other quality assurance tasks involve assessing the image quality of the reconstructed CT datasets used for target delineation, radiation dose calculation, and image registration. Key documents that highlight guidelines for the safe implementation of 4D‐CT into a radiotherapy clinic include the report of the AAPM Task Group 66,^8^ the report of the AAPM Task Group 76,^13^ and the Health Canada Safety Code 35.^10^

## 3 RELATED TECHNICAL QUALITY CONTROL GUIDELINES

In order to comprehensively assess computed tomography simulator performance, additional guideline tests, as outlined in related CPQR Technical Quality Control (TQC) guidelines must also be completed and documented, as applicable. Related TQC guidelines, available at cpqr.ca, include:

1. Safety Systems
2. Major Dosimetry Equipment

## 4 TEST TABLES

Tables 1, 2, 3, 4 present the daily, monthly, quarterly, and annual recommended tests, which are described below.

**Table 1:** Daily quality control tests

Designator	Test	Action
Daily		
D1	Lasers (alignment, spacing, motion)	±1 mm
D2	CT number for water – mean (accuracy)	0 ± 4 HU
D3	CT number for water – standard deviation (noise)	Reproducible (±10% or 0.2 HU from baseline value, whichever is larger)
D4	CT number for water – mean vs. position (uniformity)	±2 HU
D5	Respiratory monitoring system	Functional
D6	Audio/video coaching systems (if applicable)	Functional

**Table 2:** Monthly quality control tests

Designator	Test	Action
Monthly		
M1	Couch tabletop level	2 mm over the length and width of the tabletop
M2	Lasers (orthogonality/orientation)	±1 mm over the length of laser projection
M3	Couch displacement	±1 mm

**Table 3:** Quarterly quality control tests

Designator	Test	Action
Quarterly		
Q1	CT number for other materials – mean (accuracy)	Reproducible (set action level at time of acceptance)
Q2	3D low contrast resolution	Reproducible (set action level at time of acceptance)
Q3	3D high contrast spatial resolution (at 10 and 50% modulation transfer function [MTF])	Reproducible (±0.5 lp/cm or ±15% of the established baseline value, whichever is greater)
Q4	Slice thickness (sensitivity profile)	Reproducible (±1 mm from baseline for slices ≥2 mm, ±50% from baseline for slices of 1 to 2 mm, ±0.5 mm from baseline for slices <1 mm)
Q5	Amplitude and periodicity of surrogate with monitoring software and/or CT console	1 mm, 0.1 s
Q6	4D‐CT reconstruction	Functional
Q7	Amplitude of moving target(s) measured with 4D‐CT	<2 mm
Q8	Spatial integrity and positioning of moving target(s) at each 4D respiratory phase	2 mm (FWHM) difference from baseline measurement (increased for amplitudes larger than 2 cm)
Q9	Mean CT number and standard deviation of moving target(s) at each respiratory phase	(±10 HU) and (±10%) from baseline measurement (increased for amplitudes larger than 2 cm)
Q10	4D‐CT intensity projection image reconstruction (Avg, MIP, MinIP)	2 mm (FWHM) difference from baseline measurement (increased for amplitudes larger than 2 cm)
Q11	4D data import to treatment planning system	Functional

**Table 4:** Annual quality control tests

Designator	Test	Performance
Annually		
A1	Radiation dose (CTDI_w_)	±10% from baseline
A2	X ray generation: kVp, HVL, mAs linearity	±2 kVp, ±10% difference from baseline measurement (HVL and mAs)
A3	Gantry tilt	±0.5°
A4	4D low contrast resolution at each respiratory phase	Reproducible (set action level at time of acceptance)
A5	4D high contrast spatial resolution at each respiratory phase	Reproducible (set action level at time of acceptance)
A6	4D slice thickness (sensitivity profile) at each respiratory phase	Reproducible (set action level at time of acceptance)
A7	Simulated planning	±2 mm
A8	Records	Complete
A9	Independent quality control review	Complete

Notes on Daily Tests

**Table:** 

D1	Alignment of lasers should minimally match the tolerance set for those in the treatment delivery rooms. The daily laser test is meant to ensure that the gantry lasers accurately identify the scan plane within the gantry opening. A simple phantom can be used to perform this test, as detailed in Mutic et al., 2003.^8^ The wall laser position with respect to the imaging plane shall be verified as this distance is used for patient localization marking. Finally, the accuracy of wall and ceiling laser motion shall be checked daily using displacement values within the full range of laser motion. This latter test can be simply performed with a ruler.
D2	The mean CT number of water shall be checked using a typical CT simulation protocol and a cylindrical water phantom, using a large region of interest (ROI).^10^ The protocol used for the test should alternate to cover all kVp used clinically if applicable. The action level defined for this test is the one recommended in Safety Code 35.^10^
D3	The standard deviation of CT numbers of water shall be checked using a typical CT simulation protocol and a cylindrical water phantom, using a large ROI located at the center of the phantom.^10^ The protocol used for the test should alternate to cover all kVp used clinically if applicable. The action level defined for this test is the one recommended in Safety Code 35.^10^
D4	The deviation of the mean CT number in any off‐center ROI shall be checked against the mean CT number of a ROI at the center of a cylindrical water phantom. ROIs having a diameter representing approximately 10% of the phantom's diameter^10^ located at 12, 3, 6, and 9 hr at the periphery are recommended. The protocol used for the test should alternate to cover all kVp used clinically if applicable. The action level defined for this test is the one recommended in Safety Code 35.^10^
D5	The respiratory monitoring system configuration varies from center to center. For those using a third‐party monitoring system, ensure the external surrogate is visible on any in‐room monitor and its motion is being tracked and recorded by the monitoring software. Also, ensure that the interface between the monitoring software and the CT is functional. Also, ensure that all applicable network drives from workstations containing the monitoring software are mapped to the CT console before CT acquisition.
D6	Ensure any audio/video coaching software is functioning properly. Although it is recommended that this test is performed daily, it is reasonable to perform on days of use only.

Notes on Monthly tests

**Table:** 

M1	The CT scanner tabletop should be level and orthogonal with the imaging plane. This test shall be performed radiographically as a level will provide readings relative to a horizontal reference and not to the imaging plane. A detailed procedure is available in Mutic et al., 2003.^8^ If the scanner is used for diagnostic radiology purposes, this test shall be performed monthly or whenever the flat tabletop is removed.
M2	The gantry, wall, and ceiling lasers shall be parallel and orthogonal with the imaging plane over the full length of laser projections. A detailed procedure to perform these tests is available in Mutic et al., 2003.^8^
M3	The table vertical and longitudinal motion according to digital indicators shall be accurate and reproducible. This test can be simply performed with a long ruler, as detailed in Mutic et al., 2003.^8^ This test shall be performed with a typical patient load (≈80 kg).

Notes on Quarterly tests

**Table:** 

Q1–4	CT image performance is highly dependent on the scan technique used. These tests should be conducted for typical oncology protocols, for all kVp used clinically. Action levels should be developed locally depending on the equipment available. Routine monitoring of these parameters should be based on performance at installation.
Q5	The ability of the respiratory monitoring system to accurately monitor the motion of an external surrogate is crucial for ensuring 4D‐CT reconstruction integrity. For systems that use external marker blocks, the amplitude and periodicity of the external block should be performed with a programmable respiratory motion phantom (e.g., Quasar^TM^ Respiratory Motion Phantom, Modus Medical Devices, London, Canada). The phantom must contain a target of known geometry and with enough contrast to surrounding static portions of the phantom to be visualized on CT and must be compatible with the external surrogate used for clinical 4D‐CT reconstruction. The monitoring software must be able to calculate accurately the amplitude of the external surrogate. At minimum, a single amplitude within typical clinical range (e.g., 1–2 cm peak‐to‐peak) is required, but varying amplitudes allow for a more comprehensive test. The same applies to varying periodicity of the phantom. Motion in the superior/inferior direction only is permitted. However, motion of the target in all 3 dimensions allows for a more comprehensive test as long as the 3D trajectory is known. The action level defined for this test must be within 2 mm and the known respiratory motion period within 0.1 s. For systems that use a bellows device or Anzai belt, ensuring functionality (e.g., checking for leaks in the bellows device) and reproducibility of the signal is required.
Q6	For each 4D‐CT protocol used clinically, ensure that the console software reconstructs the data into the appropriate number of respiratory phases, each containing the same number of axial slices.
Q7	The amplitude of the internal target must be measured using the 4D‐CT datasets. This can be accomplished by using appropriate imaging grid tools or by calculating the centroid motion of the internal target(s). The action level defined for this test must be within 2 mm of known amplitude.
Q8	The geometry, including the target diameter, as well as the location of the target at all respiratory phases should be reproducible. The diameter can be calculated either using the grid tools or by a centrally located line profile in the direction of target motion and perpendicular to the target motion, where the full‐width‐half‐maximum value (FWHM) can be extracted. The location of the target at all phases can be calculated using on console grid tools. The action level defined for this test must be within 2 mm of those established at acceptance. The tolerance can be increased for amplitudes greater than 2 cm.
Q9	The mean CT number of the moving target(s) shall be checked using standard CT simulation protocols at each phase of the respiratory cycle. This should be performed for each 4D‐CT protocol used clinically. Also, the mean CT number must not vary significantly across all respiratory phases. The standard deviation of CT numbers of the moving target shall be checked at all phases of the respiratory cycle using either a 2D‐ROI representing at least 40% of the target diameter located near the target center or a 3D‐ROI representing at least 40% of the target volume. The recommended action level defined for these tests are (±10 HU) from the mean CT number measured at acceptance and (±10%) of the standard deviation measured at baseline. The tolerance can be increased for amplitudes greater than 2 cm.
Q10	Any post processed image creation used for radiation treatment planning using 4D‐CT images should be tested. This includes the creation of time averaged CT images, maximum intensity projection (MIP) images, and minimum intensity projection images (MinIP). This can be verified by using the on console grid tool and line profile to measure the diameter of the target and the expected CT number variation in the direction of motion. The action level defined for this test must be within 2 mm of those established at acceptance. The tolerance can be increased for amplitudes greater than 2 cm.
Q11	Successful export of the 4D‐CT dataset into the treatment planning system must be demonstrated.

Notes on Annual tests

**Table:** 

A1	CTDI_w_ should be measured over a clinically relevant range. Action levels are with respect to baseline CTDI_w_ measured at the time of commissioning. Ideally, the baseline values will be within ±10% of the manufacturers specifications, as recommended in Safety Code 35,^10^ although it is recognized that this may not be achievable on current clinical systems.Dose measurements should be performed annually or after tube replacement or servicing to validate the kVp and mAs for each 4D‐CT reconstruction technique used clinically. For centers that have a Philips Big Bore Brilliance CT scanner, the mA varies with pitch to ensure the total imaging dose is the same for equal scan lengths. In such cases, dose measurements should be performed for a range of pitches used clinically. The Unfors Raysafe Xi System (Raysafe, Billdal, Sweden) is one example of a system that can simultaneously measure kVp, mAs, and dose. When testing 4D protocols, it is not required for this system to be moving. A simple motion phantom that drives 4D‐CT reconstruction may be used. Half value layer (HVL) and CT dose index (CTDI_w_) should be measured over a clinically relevant range. Action levels are with respect to baseline HVL and CTDI_w_ measured at the time of commissioning. Ideally, the baseline values will be within ±10% of the manufacturer's specifications, as recommended in Safety Code 35.
A2	kVp and HVL should be measured over a clinically relevant range. Routine monitoring of these parameters should be based on performance at installation and manufacturer's specifications.
A3	The gantry tilt shall be 0° for radiation therapy applications. The digital gantry angle readout shall be verified using a level for gantry 0°. Additionally, it shall be checked that the gantry accurately returns to its nominal position after tilting. This test shall ideally be performed during a quarterly preventative maintenance inspection with the CT cosmetic cover removed. It is the responsibility of the CT personnel to make sure than the gantry tilt is 0° before any CT simulation exam. Ideally, a CT dedicated exclusively to radiation oncology simulation should not allow scans when the gantry is tilted.
A4–6	4D‐CT image performance is highly dependent on the protocol used. These tests should be conducted for each kVp and mAs used clinically, as well as for each 4D‐CT reconstruction technique used clinically (time‐based, phase‐based, or amplitude‐based). Ideally, this can be accomplished by using CT‐QA phantoms, such as the CATPHAN^®^ (The Phantom Laboratory, Salem, USA), that can be motion driven (e.g., CATPHAN Shaker, Modus Medical Devices, London, Canada). However, an acceptable alternative is to use a simple motion phantom to drive 4D‐CT reconstruction, but keeping the CT‐QA phantom static. An alternative phantom could include a customized insert to an already existing programmable respiratory motion phantom that can capture the same imaging metrics as the CATPHAN. Action levels should be developed locally. Annual monitoring of these parameters should be based on performance at installation.
A7	To verify the complete CT simulation process, it is recommended that a simulated planning test be part of a quality assurance program. A phantom with various markers can be scanned with a CT simulation protocol; the images transferred and virtually simulated, and marked with the lasers according to the laser/couch output data.
A8	Documentation relating to the daily quality control checks, preventive maintenance, service calls, and subsequent checks shall be complete, legible, and the operator identified.
A9	To ensure redundancy and adequate monitoring, a second qualified medical physicist shall independently verify the implementation, analysis, and interpretation of the quality control tests at least annually. This verification shall be documented.

## CONFLICT OF INTEREST

All authors approved the final manuscript, and declared that they have no potential conflicts of interest to this work.
